# Supplementary material for: Integrating simultaneous interfacial shear rheology with neutron reflectometry for structural and dynamic analysis of fluid interfacial systems
Source: J Appl Crystallogr. 2026 Apr 22;59(Pt 3):751–64. doi: 10.1107/S1600576726002104 (PMC13224800; doi:10.1107/S1600576726002104)
Supplement: Supplementary file 1 [file j-59-00751-sup1.pdf]

JOURNAL OF APPLIED CRYSTALLOGRAPHY

Volume 59 (2026)

Supporting information for article:

Integrating simultaneous interfacial shear rheology with neutron reflectometry for structural and dynamic analysis of fluid interfacial systems

Pablo Sánchez-Puga, Javier Tajuelo, Javier Carrascosa-Tejedor, Miguel Ángel Rubio, Philipp Gutfreund and Armando Maestro

Supporting Information of: "Integrating simultaneous ISR with NR for Structural and Dynamic Analysis of fluid Interfacial Systems"

Pablo Sánchez-Puga [† ‡ a], Javier Tajuelo [b], Javier Carrascosa-Tejedor [a], Miguel Ángel Rubio [c], Philipp Gutfreund [a], and Armando Maestro [d,e]

[a]Institut Laue-Langevin, 38042, Grenoble (France)

[b]Departamento de Física Interdisciplinar, Facultad de Ciencias, Universidad Nacional de Educación a Distancia (UNED), 28232, Las Rozas (Spain)

[c]Departamento de Física Fundamental, Facultad de Ciencias, Universidad Nacional de Educación a Distancia (UNED), 28232, Las Rozas (Spain)

[d]Centro de Física de Materiales (CFM-MPC), CSIC-EHU, Paseo Manuel de Lardizabal 5, 20018, Donostia-San Sebastián (Spain)

[e]IKERBASQUE-Basque Foundation for Science, Bilbao (Spain)

[†]Corresponding author.

[‡]Present address: Department of Physics and Astronomy, Uppsala University, Box 516, Uppsala S-751 20, Sweden.

1 Estimation of the uncertainty of rheological measurements

Estimating uncertainty in interfacial shear rheology measurements typically involves considering the effects of two very different sources of uncertainty. On the one hand, there is instrumental error, that is, the uncertainty coming from the instrument and the procedures for the analysis of the output data. However, there is uncertainty as a result of poor experimental repeatability that can be attributed to the variability of samples or the spreading method. In this section, we will only consider instrumental errors because the repeatability problem depends strongly on the samples and is typically dealt with by averaging the results of several repetitions of each experiment, a procedure that is usually difficult to implement when NR measurements are performed because of the availability and cost of deuterated materials and beam time awarded. Consequently, in this section we study the noise floor and resolution for interfacial shear rheology measurements.

1.1 The rheology signals and their Fourier transforms

The extraction of the amplitude and phase of the torque and angular displacement signals, and their uncertainties, is carried out by Fourier transformation of the experimental signals (Klein *et al.*, 2019; Renggli *et al.*, 2020). The signals are digitised arrays of data that represent the angular displacement, $\phi(t)$ and the torque, $M(t)$, imposed by the rheometer, in the strain control

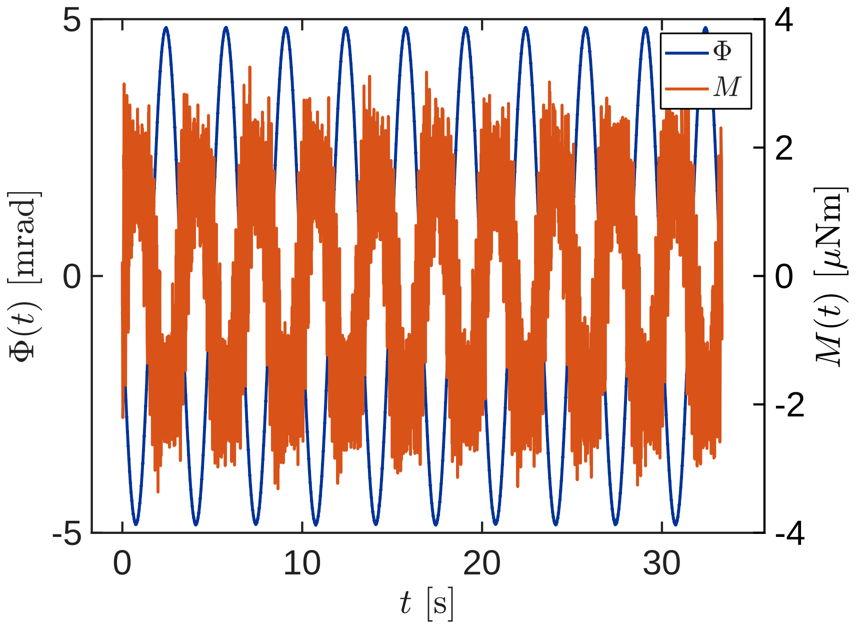

Figure S1: Example of two typical time signals at $f = 0.3$ Hz and $\gamma_s = 3$ %, showing a clean $\phi(t)$ signal (blue line) and a noisy $M(t)$ signal (red line).

mode, on the sample.

The signals are digitised so that the data are equally spaced in time. Typically, the experimental signals are digitised waveforms that contain 20 to 30 periods of the oscillation. Only the last 8-10 periods are retained for the Fourier transformation, so that any possible transients have faded away. Using an integer number of periods of the oscillation frequency minimises the effects of the rectangular time window on the spectra.

In Figure S1 we show typical signals corresponding to the angular displacement, $\phi(t)$ and the torque $M(t)$ imposed by the rheometer on the sample. These particular signals were obtained for an oscillation frequency, $f = 0.3$ Hz and a small strain, $\gamma_s = 3$ % and under typical sampling conditions (sampling time, $\tau = 33.2107$ s and $\Delta\tau = 10^{-4}$ s). The torque signal visibly contains more high frequency noise than the angular displacement signal. Torque signals with high-frequency noise usually appear in rotational rheometers when working in strain control mode in regimes dominated by the inertia of the probe+rotor ensemble, which demand very low torque amplitudes.

MATLAB standard 'fft' routines have been used to compute the Discrete Fourier Transform (DFT). Then, the signal amplitude (modulus of the Fourier transform at the main peak) and phase (arc tangent of the ratio between the imaginary and real parts of the Fourier transform at the main peak) can be calculated for the torque and angular position signals.

The amplitude spectra corresponding to the Fourier transformation of the typical signals shown in Figure S1 are shown in Figure S2. As expected, the noise in the angular displacement spectrum

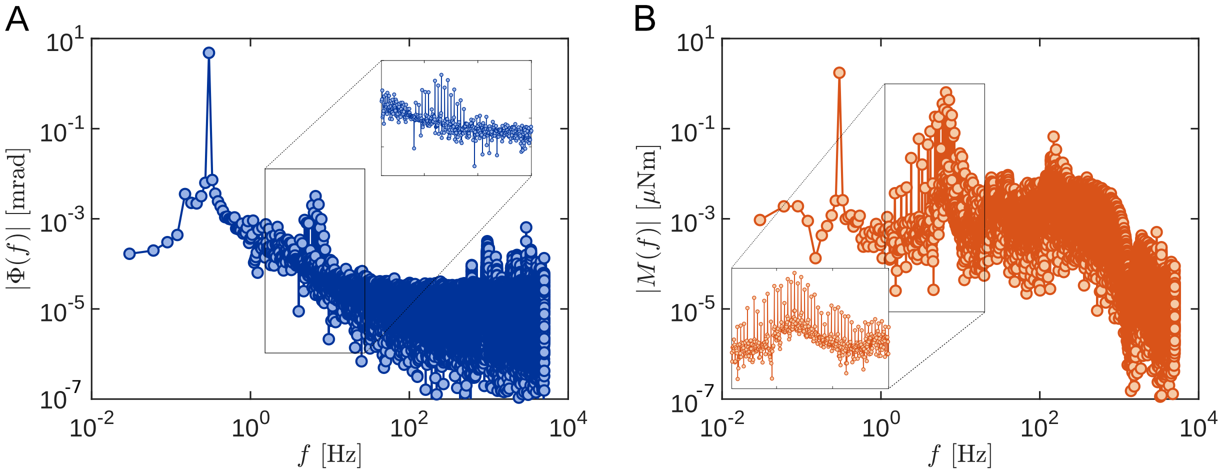

Figure S2: Amplitude spectra of the angular displacement (panel A) and torque signals (panel B) shown in Figure S1.

is significantly lower than in the torque spectrum. High-frequency noise has a peculiar structure in both signals (see insets) consisting of a comb of equally spaced peaks separated by a frequency interval equal to the frequency of the main peak. This noise is most likely induced by the control loop that rules the strain control mode of the instrument.

However, in all the measurements reported here, the peak at the frequency f_0 in the torque amplitude spectra is always outside the high amplitude noise bandwidth. Interestingly, although the main components of the large high-frequency noise (around $f_n \sim 40$ Hz) that appears in the torque spectra does not affect the shape or position of the main peak. The reason is that the regime is inertia dominated, i.e. the probe + rotor inertia induces a f^{-2} cut-off that filters out higher frequency components. This can be checked in Figure S2A and B, where the ratio between the signal and the noise peak amplitudes is $\frac{\Phi(f_n)}{\Phi(f_0)} \sim \frac{M(f_n)}{M(f_0)} \left(\frac{f_n}{f_0}\right)^{-2}$. In other words, the high-frequency noise in the torque is transmitted to the sample largely attenuated and, therefore, it does not affect the angular displacement and, consequently, neither the rheological measurements.

1.2 Estimating the uncertainty

Estimation of uncertainty in the parameters of sinusoidal signals with noise is a delicate problem. Regarding the signals coming from rotational rheometers, the problem has been faced recently for both bulk (Singh *et al.*, 2019) and interfacial (Renggli *et al.*, 2020) rheometry. Mathematical results based on the Cramer-Rao criterion (Kay, 1993; Stoica *et al.*, 2005), yield expressions for variances in the estimators of sinusoidal parameters. Let us consider a sinusoidal signal with additive white Gaussian noise, described by

$$x_i(n) = A_i \cos(2\pi f_0 n + \varphi_i) + w_i(n), \quad n = 1, 2, ...N, \tag{1}$$

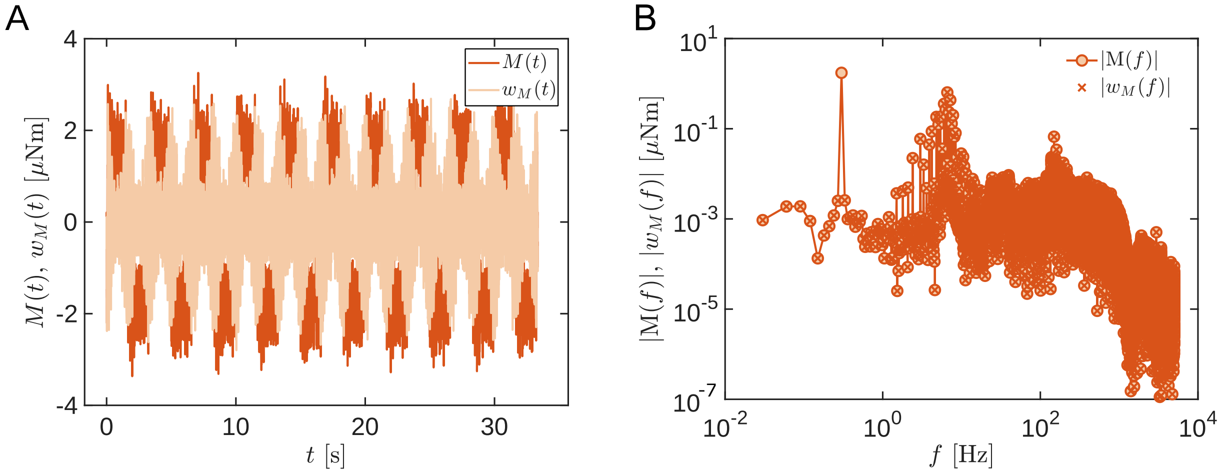

Figure S3: Panel A: Torque (dark line) and torque's noise (light line) signals corresponding to the data shown in Figure S1. Panel B: Amplitude of the DFT of the signals. The spectra of the full signal and the noise differ just at the driving frequency, f_0, where almost null amplitude ($\sim 10^{-20}$) is found in the noise spectrum (point not shown)

where A_i and φ_i are the amplitude and phase of cosine, f_0 the frequency in Hz, $w_i(n)$ an additive noise, with 0 mean and variance σ_i^2, N the total amount of data and $i = M$ and Φ for torque and angular displacement, respectively. The variances of the estimators corresponding to the amplitude and phase of the oscillatory signals are (Kay, 1993)

$$\sigma^2(A_i) = \frac{2}{N}\sigma_i^2, \tag{2}$$

$$\sigma^2(\varphi_i) = \frac{4(2N-1)}{N(N-1)}\frac{\sigma_i^2}{A_i^2}. \tag{3}$$

The angular displacement signals show very low noise that can be well described by an additive white Gaussian noise. Hence, the expressions above can be readily applied to angular displacement signals. However, the torque signals shown here are better described as sinusoids plus an additive band-limited Gaussian noise, as shown in Figs. S3a and b, where we plot, respectively, the torque signal, $M(n)$, and its noise, $w_M(n) = x(n) - A_M \cos(2\pi f_0 n + \varphi_M)$, in panel A, and the amplitude spectra of the torque signal, $|M(f)|$, and its noise, $w_M(n)$, namely, $|W_M(f)|$.

Then, we can use the above expressions just taking into account (Kay, 1993) the contribution of the noise at frequency f_0, that is, substituting σ^2 by $|W(f_0)|^2$ in expressions (2) and (3), then yielding

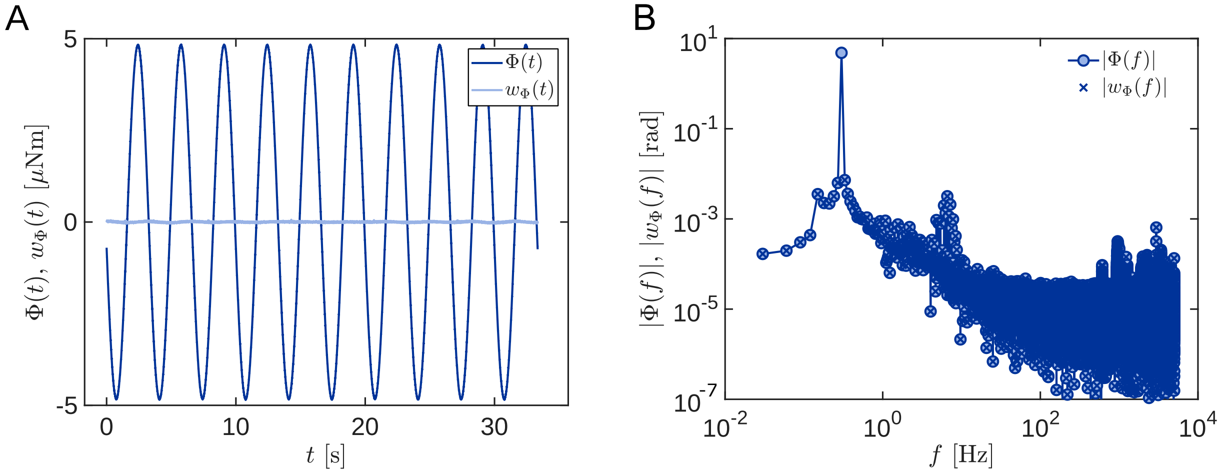

Figure S4: Panel A: Angular displacement (dark line) and angular displacement's noise (light line) signals corresponding to the data shown in Figure S1. Panel B: Amplitude of the DFT of the signals. The spectra of the full signal and the noise differ just at the driving frequency, f_0, where almost null amplitude ($\sim 10^{-20}$) is found in the noise spectrum (point not shown).

$$\sigma^2(A_i(f_0)) = \frac{2}{N}|W_i(f_0)|^2, \tag{4}$$

$$\sigma^2(\varphi_i(f_0)) = \frac{4(2N-1)}{N(N-1)}\frac{|W_i(f_0)|^2}{A_i(f_0)^2}. \tag{5}$$

Now, $|W_i(f_0)|^2$ can be estimated using as a safe upper bound the average of the DFT values at the bins adjacent to f_0, i. e.,

$$|W_i(f_0)| = \frac{1}{2}\left[|W_i(f_0+\delta f)| + |W_i(f_0-\delta f)|\right], \tag{6}$$

where δf is the bin width in the DFT.

Such a procedure yields a convenient and automatic method for estimating the uncertainties in the amplitude and phase values of these experimental signals. Then we may estimate the uncertainties of the amplitudes and phases of both signals as follows.

$$\Delta\Phi(f_0) = \sqrt{\sigma^2(\Phi(f_0))} = \sqrt{\frac{2}{N}}|W_\Phi(f_0)|, \tag{7}$$

$$\Delta\varphi_\Phi(f_0) = \sqrt{\sigma^2(\varphi_\Phi(f_0))} = 2\sqrt{\frac{2N-1}{N(N-1)}\frac{|W_\Phi(f_0)|}{\Phi(f_0)}}, \tag{8}$$

$$\Delta M(f_0) = \sqrt{\sigma^2(M(f_0))} = \sqrt{\frac{2}{N}}|W_M(f_0)|, \tag{9}$$

$$\Delta\varphi_M(f_0) = \sqrt{\sigma^2(\varphi_M(f_0))} = 2\sqrt{\frac{2N-1}{N(N-1)}\frac{|W_M(f_0)|}{M(f_0)}}. \tag{10}$$

Finally, the modulus and phase of the complex amplitude ratio, $AR^*(f_0)$, are

$$|AR^*(f_0)| = \frac{|M(f_0)|}{|\Phi(f_0)|}; \quad \text{and} \quad \varphi(f_0) = \varphi_M(f_0) - \varphi_\Phi(f_0). \tag{11}$$

Then, again applying quadratic propagation of errors, the absolute error in the modulus of the complex amplitude ratio will be

$$\Delta|AR^*(f_0)| = \frac{|M(f_0)|}{|\Phi(f_0)|}\sqrt{\left(\frac{\Delta M(f_0)}{M(f_0)}\right)^2 + \left(\frac{\Delta\Phi(f_0)}{\Phi(f_0)}\right)^2} \tag{12}$$

On the other hand, the absolute error of the phase of the complex amplitude ratio will be

$$\Delta\varphi(f_0) = \sqrt{(\Delta\varphi_M(f_0))^2 + (\Delta\varphi_\phi(f_0))^2} \tag{13}$$

Two ways can be considered regarding the estimation of the error in $G_s^*(f_0)$ due to the error in $AR^*(f_0)$. The first and probably the most accurate way to estimate the error propagation from $AR^*(f_0)$ to $G_s^*(f_0)$ is to study numerically how the square in the complex plane defined by $AR^*(f_0)$ and its uncertainties maps to the complex plane G_s', G_s'' using the FFBDA package (Sánchez-Puga *et al.*, 2021; Sanchez-Puga & Rubio, 2025). This procedure involves nine applications of the iterative scheme for each data point that, depending on the computer at hand, might be too time-consuming for an on-the-fly implementation of the computation during the experiments. However, the error bars for the results shown in Figures 4, 5 and 7 of the main document were obtained by this procedure.

Second, a much simpler and faster, but probably less accurate, method would be to just propagate the error in the values of AR^*_{exp} through the expression used to obtain the values of G_s^*, that

is,

$$G_s^* = \frac{-AR_{\text{exp}}^* - I\omega^2 - AR_1^*(g_1^*) - AR_2^*(g_2^*)}{2\pi R_6^2 \left({\bar{R}_5}^3 \frac{\partial}{\partial \bar{r}} \left(\frac{g_s^*}{\bar{r}} \right) \Big|_{\bar{r}=\bar{R}_5} - \frac{\partial}{\partial \bar{r}} \left(\frac{g_s^*}{\bar{r}} \right) \Big|_{\bar{r}=\bar{R}_6} \right)}. \tag{14}$$

It is interesting to compare the results of the error estimation procedure described so far with those obtained by applying the procedure recently proposed in the literature (Singh *et al.*, 2019; Renggli *et al.*, 2020). According to Singh *et al.*(Singh *et al.*, 2019)

$$\Delta |G_s^*| = \frac{C_M}{C_\Phi} \sqrt{\left(\frac{1}{\Phi_0} \right)^2 \Delta M_0^2 + \left(\frac{M_0}{\Phi_0^2} \right)^2 \Delta \Phi_0^2}, \tag{15}$$

and, using a simple geometric construction, the error in the argument of the dynamic modulus can be estimated as

$$\Delta \delta_s = \sin^{-1} \left(\frac{\Delta M_0}{M_0} \right) + \sin^{-1} \left(\frac{\Delta \Phi_0}{\Phi_0} \right). \tag{16}$$

As $G_s^* = |G_s^*| e^{i\delta_s}$, we can write

$$\Delta G_s' = \sqrt{(\cos \delta_s \; \Delta |G_s^*|)^2 + (-|G_s^*| \sin \delta_s \; \Delta \delta_s)^2}, \tag{17}$$

$$\Delta G_s'' = \sqrt{(\sin \delta_s \; \Delta |G_s^*|)^2 + (|G_s^*| \cos \delta_s \; \Delta \delta_s)^2}. \tag{18}$$

Given that the method based on the numerical error propagation through the FFBDA scheme contains less simplifying assumptions and, therefore, should better represent the data uncertainty, consequently, all errors considered here and in the main publication have been estimated using numerical error propagation through the FFBDA scheme.

The errors estimated using numerical error propagation through the FFBDA algorithm or expressions (15) and (16) in the data shown in Figure 5 of the main document yield error bars smaller than the size of the symbol. For the sake of completeness, we illustrate the relative errors in the dynamic moduli, $\delta G_s' = \Delta G_s'/G_s'$, and $\delta G_s'' = \Delta G_s''/G_s''$, for such data in Fig. S5. Evidently, the results obtained from both methods have a very similar appearance, although the values obtained through the method proposed by Singh *et al.* (Singh *et al.*, 2019) are consistently lower by approximately a factor of 2.

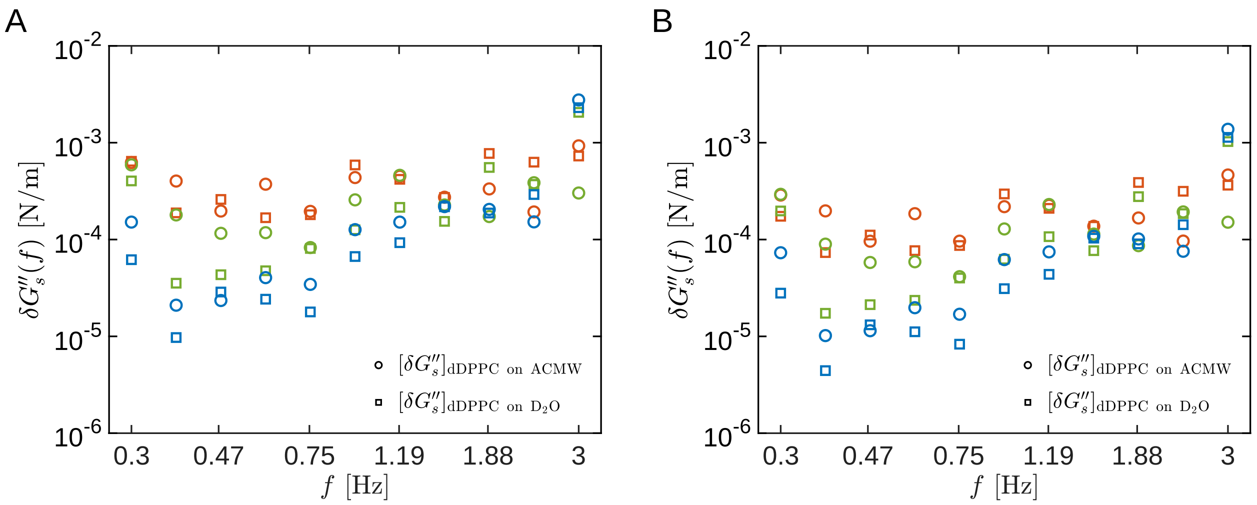

Figure S5: Loss modulus relative errors, $\delta G''_s(f)$, as a function of frequency, at $\gamma_s = 3$ %, obtained through: panel A numerical error propagation in the FFBDA, and panel B using expressions (15) and (16). dDPPC monolayers on ACMW subphase (circles) and on D_2O subphase (squares). Triangles: apparent loss modulus for a clean air/water interface. Red symbols: $\Pi = 25$ mN/m. Green symbols: $\Pi = 30$ mN/m. Blue symbols: $\Pi = 45$ mN/m.

2 Simple second order dynamic model for a torsion rheometer under strain control

Let us assume that we are working in a high Bq^* regime, so that the drags of the bulk fluid phases are negligible and the interface drag can be represented as $T_s = C_g \eta_s^* \dot{\theta}$, where C_g is a geometric coefficient and $\eta_s^* = \eta_s' - i\eta_s''$. Then, in the strain controlled mode, the dynamics of the rotor+probe ensemble can be represented by the equation

$$I\ddot{\theta}^*(t) + C_g \eta_s^* \dot{\theta}^*(t) = T^*(t). \tag{19}$$

Assuming also that $\theta^*(t) = \theta_0 e^{i\omega t}$ and $T^*(t) = T_0 e^{i(\omega t + \delta)}$, we get

$$-I\omega^2 \theta_0 + iC_g \omega \eta_s^* \theta_0 = T_0 e^{i\delta}, \tag{20}$$

or

$$-I\omega^2 + iC_g \omega \eta_s^* = AR^*_{exp}. \tag{21}$$

Hence,

$$|AR^*_{exp}| = \sqrt{(C_g \eta_s'' \omega - I\omega^2)^2 + (C_g \eta_s' \omega)^2}, \tag{22}$$

or, equivalently,

$$|AR^*_{exp}| = \sqrt{(C_g G'_s - I\omega^2)^2 + (C_g G''_s)^2}, \tag{23}$$

and

$$\delta = \arctan\left(\frac{C_g \eta'_s \omega}{C_g \eta''_s \omega - I\omega^2}\right), \tag{24}$$

or, equivalently,

$$\delta = \arctan\left(\frac{C_g G''_s}{C_g G'_s - I\omega^2}\right), \tag{25}$$

3 Reflectivity curves

For completeness in Figure S6 we show the reflectivity curves corresponding to $\Pi = 35$ and 45 mN/m for the two isotopic contrasts used, along with their respective fitting lines.

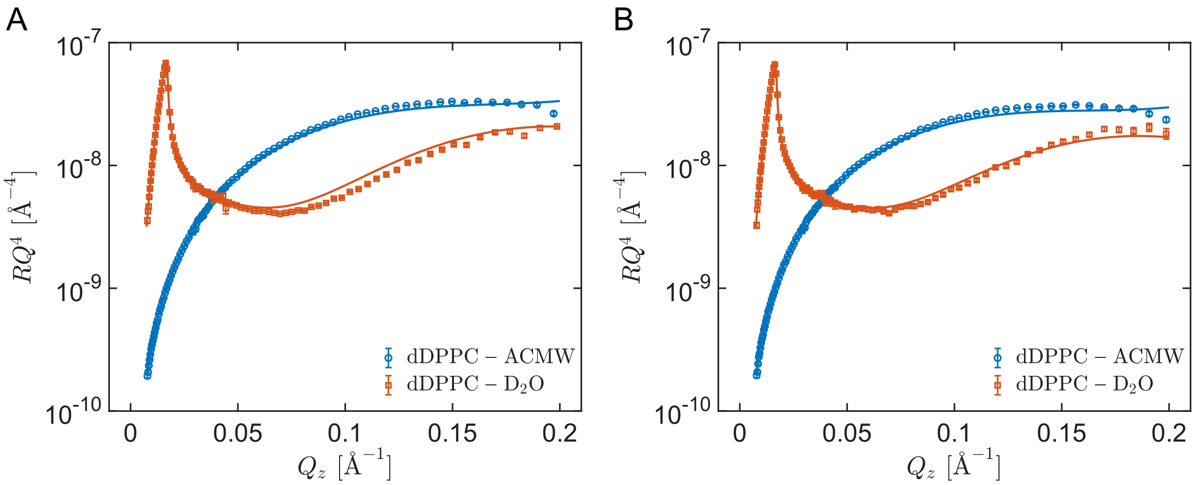

Figure S6: RQ^4, as a function of the vertical scattering vector, Q_z. Panel A at $\Pi = 35$, and panel B at $\Pi = 45$ mN/m for the two different isotopic subphases used here.

References

Kay, S. M. (1993). *Fundamentals of statistical signal processing: estimation theory.* Prentice-Hall, Inc.

Klein, C. O., Theodoratou, A., Rühs, P. A., Jonas, U., Loppinet, B., Wilhelm, M., Fischer, P., Vermant, J. & Vlassopoulos, D. (2019). *Rheologica Acta*, **58**, 29–45.

Renggli, D., Alicke, A., Ewoldt, R. H. & Vermant, J. (2020). *Journal of Rheology*, **64**, 141–160.

Sanchez-Puga, P. & Rubio, M. A. (2025). *Computer Physics Communications*, **310**, 109499. https://www.sciencedirect.com/science/article/pii/S0010465525000025

Sánchez-Puga, P., Tajuelo, J., Pastor, J. M. & Rubio, M. A. (2021). *Advances in Colloid and Interface Science*, **288**, 102332. https://www.sciencedirect.com/science/article/pii/S0001868620306011

Singh, P. K., Soulages, J. M. & Ewoldt, R. H. (2019). *Rheologica Acta*, **58**(6), 341–359.

Stoica, P., Moses, R. L. *et al.* (2005). *Spectral analysis of signals*, vol. 452. Pearson Prentice Hall Upper Saddle River, NJ.
